# Get in touch with numbers – an approximate number comparison task in the haptic modality

**DOI:** 10.3758/s13414-021-02427-6

**Published:** 2022-01-21

**Authors:** Marco Carlo Ziegler, Knut Drewing

**Affiliations:** 1 University of Giessen, Department of Psychological Assessment, Giessen, Germany; 2 University of Giessen, Department of Experimental Psychology - HapLab, Giessen, Germany

**Keywords:** Multisensory processing, Haptics, Visual perception

## Abstract

The Approximate Number System (ANS) is conceptualized as an innate cognitive system that allows humans to perceive numbers of objects or events (>4) in a fuzzy, imprecise manner. The representation of numbers is assumed to be abstract and not bound to a particular sense. In the present study, we test the assumption of a shared cross-sensory system. We investigated approximate number processing in the haptic modality and compared performance to that of the visual modality. We used a dot comparison task (DCT), in which participants compare two dot arrays and decide which one contains more dots. In the haptic DCT, 67 participants had to compare two simultaneously presented dot arrays with the palms of their hands; in the visual DCT, participants inspected and compared dot arrays on a screen. Tested ratios ranged from 2.0 (larger/smaller number) to 1.1. As expected, in both the haptic and the visual DCT responses similarly depended on the ratio of the numbers of dots in the two arrays. However, on an individual level, we found evidence against medium or stronger positive correlations between “ANS acuity” in the visual and haptic DCTs. A regression model furthermore revealed that besides number, spacing-related features of dot patterns (e.g., the pattern’s convex hull) contribute to the percept of numerosity in both modalities. Our results contradict the strong theory of the ANS solely processing number and being independent of a modality. According to our regression and response prediction model, our results rather point towards a modality-specific integration of number and number-related features.

## Introduction

To be able to perceive and process numbers of events or objects, humans as well as other animals require a neuronal grounding. A commonly accepted idea is that humans are equipped with an innate number sense that allows processing of numerosity (Butterworth, 2010; Dehaene, 2011; Feigenson et al., 2004; Piazza, 2010). Numerosity (also known as cardinality) is defined by Nieder (2016) as the number of countable items in a given set (in contrast to, e.g., discrete numerical symbols, such as Hindu-Arabic numerals). There is an ongoing debate of the exact definition of the number sense and what abilities it includes (Jordan et al., 2006; Szűcs & Myers, 2017). One established theory, the two-component model, distinguishes the number sense into one system that processes small nonsymbolic numbers of distinct individuals that fall into subitizing range (i.e., smaller than five items) and another system for larger numbers beyond four (Dehaene, 2011; Feigenson et al., 2004; Hyde, 2011; Mou & vanMarle, 2014; Olsson et al., 2016; De Smedt et al., 2013). The latter one is often denoted as the Approximate Number System (ANS), a cognitive system that enables people “to represent quantities as imprecise, noisy mental magnitudes without verbal counting or numerical symbols” (Park & Brannon, 2013, p. 1). A signature of the ANS is ratio dependence (e.g., when humans compare two nonsymbolic numerosities; Brannon & Merritt, 2011). Performance in such comparison tasks is determined by the ratio of the two numbers and not their numerical difference. For instance, the comparison performance between 5 and 6 dots is similar to that between 10 and 12 dots, and it is faster and more accurate than that between 7 and 8 dots (Halberda et al., 2008). Ratio dependence results from fuzzy mental representations of larger numbers and is consistent with Weber´s Law (Brannon & Merritt, 2011; Feigenson et al., 2004; Szkudlarek & Brannon, 2017). Another core assumption of the ANS is that numerosity is represented in an abstract format that is shared across sensory modalities (Barth et al., 2005; Feigenson et al., 2004).

Whereas ratio dependence of approximate numerosity has been demonstrated repeatedly (Feigenson et al., 2004; Mou & vanMarle, 2014), the assumption of a truly abstract representation of number is topic of an ongoing debate (Anobile et al., 2018; Anobile et al., 2016; Barth et al., 2003; Barth et al., 2005; Gebuis et al., 2016). In the present study, we examined the ability to compare approximate numerosities in the visual and the haptic modality and investigated their commonalities and differences.

The ANS theory suggests that of any given stimulus material (e.g., a dot pattern) numerosity gets extracted by filtering out the irrelevant confounding features of the stimuli (like varying diameter of the dots) and then accumulating the numerosity (Brannon & Merritt, 2011; Dehaene, 2011; Dehaene & Changeux, 1993; Gebuis et al., 2016), implying abstract representations that are independent of the used sensory modality. Barth et al. (2003) stated that a truly abstract number sense should be robust against variation of stimulus presentation formats and actual presented stimulus material. Comparing performances of number estimation tasks between different modalities as well as cross-modal paradigms are a straightforward way to examine these assumptions.

Research on larger numerosities has mainly been dedicated to the visual modality whereas other modalities have not (yet) received similar attention. At least a few studies have utilized auditory stimulus material, e.g., tone sequences in audiovisual habituation paradigms with newborns (Izard et al., 2009), or sequential dot and tone sequences in same–different or arithmetic tasks (Barth et al., 2003; Barth et al., 2005). The assumption of a system that is capable of extracting numerosity independently of the sensory input has been addressed by Barth et al. (2003). They distinguished between “not modality specific” representation of number and a modality specific “perceptual representation” that also might include stimulus specific influences. They argued that if a numerosity representation is specific to a modality, cross-modal numerosity comparisons must fail because modality specific percepts will not be compatible. In a series of experiments, Barth et al. (2003) tested participants in conditions varying numerosity task formats (temporal/spatial) along with sensory modality (within-modality/cross-modal): Discrimination of numerosity did not differ significantly between within-modality conditions and cross-modal conditions when both stimuli in a trial were following the same task format (within-modality: two auditory or two visual sequences; cross-modal: comparing an auditory to a visual sequence). Also, discrimination performance within a single modality did not differ when using different task formats (comparing visual sequences to spatial visual dot arrays) as compared with using one task format (two visual sequences or two visual arrays). The authors concluded that there must be a truly amodal representation of number, which is neither specific to a modality nor a task format, because in cross-modal and cross-task conditions participants’ performance did not fall behind the performance of their weaker within-modal and within-task condition, respectively.

However, in the same study by Barth et al. (2003), participants performed significantly worse in a combined cross-modal cross-task condition (comparing a visual dot array to tones in a sequence) than in either of the two corresponding within-modal same-task conditions (two visual dot arrays or two auditory sequences). This seems to indicate that the representation of number is at least not completely amodal, and that to some extent perceptual cues (conveyed by task formats) influence the percept of number. Similarly, Anobile et al. (2018) found that individual approximate numerosity performance hardly correlated between a spatial visual and a temporal auditory task (at least in adults), whereas individual performance within the temporal visual and auditory tasks showed substantial correlations in children and adults (Anobile et al., 2018). That is, differences in sensory modality and task format resulted in a lack of correlation in task performance within adult participants, which is strong evidence against an amodal numerosity representation that is completely shared between the two tasks. However, in the study by Anobile et al. (2018), it is not entirely clear how far the lack of correlation leads back to differences in the task format or in the sensory modalities.

Tokita et al. (2013) tried to isolate modality-specific factors influencing numerosity estimation by defining a stricter standardization for numerosity tasks in different sensory modalities. The authors used strictly parallel sequential visual and auditory task formats. Participants compared dots or tone sequences within a modality as well as in a cross-modal condition. Participants’ performances (Weber fractions) showed substantial differences between the visual and auditory modality (Tokita et al., 2013). The authors concluded this to be a contradictory result to the assumption of a modality-independent numerical representation system, since a truly abstract system processing numerosity independently would not allow for performance differences. In a subsequent study, Tokita and Ishiguchi (2016) extended this line of research by evaluating participants’ performance in comparing tone sequences and sequences of vibrations (via vibro-tactile devices). Here, on the other hand, they found no significant differences in participants’ performance between the two modalities, which they considered to be expected as the auditory and the haptic modality are more alike than are the visual and the auditory domain (Tokita & Ishiguchi, 2016).

Overall, results are mixed regarding the question of a modality-independent representation of numerosity. There are also differences in the interpretation of results (Gebuis et al., 2016), which partly relate to different understandings of the ANS concept following either a strong interpretation that the ANS solely extracts number, or a broader interpretation allowing that additionally other factors play some role. A direct comparison of results between studies is further complicated by a wide variety of measurement approaches for approximate numerosity (different instructions, stimulus ranges, presentation formats, etc.).

In the present study, we aim for a straightforward test to investigate the question if number representation is abstract and grounded in a shared modality-independent system, as well as for transparent data interpretation in terms of stronger and broader ANS concepts. The present study uses parallel tasks formats in the haptic and the visual modality (simultaneous presentation of two spatial dot arrays; cf. Tokita et al., 2013) and includes correlations of individual performances as a strong measure of shared representations (cf. Anobile et al., 2018). Surprisingly, the haptic modality has been left almost untouched for investigating these questions. However, spatially separated dot comparison tasks (a.k.a. “paired”-paradigm; Dietrich et al., 2015; Inglis & Gilmore, 2014), can be designed for the haptic modality—as it is the case for the visual but not the auditory modality. Therefore, a dot comparison task (DCT) format in the haptic modality can be implemented fairly similarly to the format that is typically used in the visual modality. From a theoretical perspective, this method, where participants have to decide which one of two spatially separated dot arrays contains more elements (Dietrich et al., 2015), is preferred when assessing a person’s ANS’s acuity, because this paradigm requires the least additional cognitive processes when perceiving numerosity (Dietrich et al., 2015). Pioneer work in approximate numerosity estimation tasks in the haptic modality was done by Ginsburg and Pringle (1988), who, however, used a direct numerosity estimation task and not a paradigm with simultaneously presented separated dot arrays. To our knowledge, the only adaptation of a haptic DCT with simultaneously presented and spatially separated arrays has been reported by Gimbert et al. (2016). They presented two stimulus sets (rectangular pads with raised dots) to 147 children and asked them to explore the pads simultaneously via active touch and to compare dot numerosity. Pairs of pads represented ratios from 1.1 (hardest) to 3.0 (easiest). The children additionally performed a visual DCT. The children were able to compare approximate quantities in the haptic DCT and showed typical and similar ratio dependent effects in the visual as well as in the haptic DCT. Gimbert et al. (2016) concluded that a shared mechanism (approximate number processing) accounted for their results.

However, this conclusion needs additional testing, because it is mainly based on the observation that the average haptic and visual performance both increase with higher ratios. As a number of different mechanisms would predict better performance with higher ratios, the shared average performance in visual and haptic tasks is not sufficient evidence for a common mechanism. A whole line of recent research has identified mechanisms that contribute to participants’ numerosity estimates, such as the convex hull or area density of a dot array (Bertamini et al., 2016; Clayton et al., 2015; Tomlinson et al., 2020). Our approach here is to estimate such factors that might additionally contribute to the number percept, and to find their relative weight in the haptic and visual response process. Thus, we investigate whether number is indeed the strongest influence to account for the results. We use an approach suggested by DeWind et al. (2015). DeWind et al. (2015) describe different orthogonal factors (number, spacing, and size) that contribute to the percept and the participant’s final response in a DCT (details in data analysis). Further, we aim to assess individual visual and haptic performance in DCTs rather than average group performance alone. The individual then serves as an additional factor besides ratio that influences performance and can be used to test for a shared mechanism: If the ANS is cross-modally shared, differences in individual performance should covary between the two sensory modalities. Moreover, we also want to advance some stimuli and design choices in the study of Gimbert et al. (2016), which may have been problematic.

First, we implemented sufficient spacing between dots in the haptic DCT (>11 mm based on Craig & Lyle, 2001) to allow participants to segregate dots as singular entities rather than perceiving them as an essentially not-countable texture. Second, Gimbert et al. (2016) seem to have used the same limited number of dot stimuli for every child in their study, which risks creating confounds and biases related to the specifically used dot configurations. In the present study, we hence varied the dot patterns used between sessions and participants. Third, we collect sufficient data to be able to report every tested ratio separately rather than an aggregation of different ratios into categories (“ratio bins”), and to allow for a basic check for psychometric features (e.g., a reliability estimation) in our novel implementation of a haptic DCT. Finally, we also calculated mean percentage correct and Weber fractions as ANS acuity indicators (omitted in Gimbert et al., 2016), which can be compared between modalities (see Anobile et al., 2018), to investigate if there is a shared underlying system that processes the same feature.

In the present study, we presented a visual and a haptic DCT to 71 individuals. Our haptic and visual DCT share all essential features, which makes them comparable to the extent that if number is the primary source for the percept, ANS acuity in both tasks should be highly correlated. These include the general paired paradigm (simultaneous spatial dot arrays) and identical dot ratios. Obviously, as was the case in the previous studies, also some modality-specific adaptations were necessary, which we report in detail below. We evaluated

- whether adult participants express typical ratio dependent performance in both tasks,
- the relative weight of factors that lead to the percept of numerosity by means of a regression model of numerosity perception for each modality,
- the test–retest reliability of the haptic DCT and the visual DCT, and
- whether participants’ individual and group-wise performances correlate between the haptic and the visual DCT.

## Methods

### Participants

Using G-Power (Version 3.1; Faul et al., 2009), we estimated a required sample size of 64 participants to achieve a power of .80 (α = .05) for a single-sided medium sized effect (ρ = .3) in the correlation of performance indices between sensory modalities. We recruited 71 participants (49 females, 22 males, mean age 24 years), 67 met the inclusion criteria and remained for the data analysis. All participants were healthy and had normal or corrected to normal vision. None of the participants had any injuries, unusual keratinization or scar tissue in the palm of their hands. Participants got course credit or eight euro per hour as compensation for their efforts. Participants gave written informed consent before enrolling in the study. All procedures and methods were consistent with the World Medical Association Declaration of Helsinki (2013) and were approved by the local ethics committee of Fachbereich 06 of the Justus-Liebig-University Gießen (LEK-FB 06).

### Design, setups, and procedure

In the experiment, each participant performed two variants of dot comparison tasks (DCTs), a visual and a haptic DCT. They had to decide which of two dot arrays contained a higher number of dots. We varied the number ratio of the presented dot arrays in the following steps: 2.00, 1.33, 1.20, 1.14, and 1.11 with a number of dots ranging from 5 to 20 per stimulus (cf. Halberda et al., 2008). We conducted each dot comparison in a “single” and a “double” variant (“version”). The double variant contained twice as many dots per stimulus compared with the single variant (Table 1). Furthermore, the more numerous dot patterns were equally frequent on the left and the right side of the screen. Our primary measure for individual ANS acuity was the individual Weber fraction (*w*) per modality condition (DeWind et al., 2015).

**Table 1:** Complete list of all dot comparisons and resulting number ratios for both visual and haptic DCTs including the additional applied control conditions for the visual DCT

Comparison		Number ratio	Visual condition
10 vs. 5	(single)	2.00	DSC / SAC
5 vs. 10	(single)	2.00	DSC / SAC
20 vs. 10	(double)	2.00	DSC / SAC
10 vs. 20	(double)	2.00	DSC / SAC
6 vs. 8	(single)	1.33	DSC / SAC
8 vs. 6	(single)	1.33	DSC / SAC
12 vs. 16	(double)	1.33	DSC / SAC
16 vs. 12	(double)	1.33	DSC / SAC
6 vs. 5	(single)	1.20	DSC / SAC
5 vs. 6	(single)	1.20	DSC / SAC
12 vs. 10	(double)	1.20	DSC / SAC
10 vs. 12	(double)	1.20	DSC / SAC
8 vs. 7	(single)	1.14	DSC / SAC
7 vs. 8	(single)	1.14	DSC / SAC
16 vs. 14	(double)	1.14	DSC / SAC
14 vs. 16	(double)	1.14	DSC / SAC
10 vs. 9	(single)	1.11	DSC / SAC
9 vs. 10	(single)	1.11	DSC / SAC
20 vs. 18	(double)	1.11	DSC / SAC
18 vs. 20	(double)	1.11	DSC / SAC

*Note*. DSC = average dot size control; SAC = surface area control.

Data collection consisted of two separate sessions. Each session took place in a quiet laboratory room in the faculty of psychology at the Justus-Liebig-University Gießen. We conducted the haptic and the visual DCT once per session, respectively. Haptic and visual DCTs were performed consecutively within each session. In the second session, we reversed the order of the DCTs for the participant. Whether the haptic or the visual task was first within a session was balanced between participants. We scheduled the sessions one week apart from each other (7 days test–retest interval). The visual DCT took about 30 minutes. The haptic DCT took about 50 minutes. In each DCT, the experimenter monitored the participant over the course of the experiment. The participants could pause the experiments any time to regain focus on the task.

#### Visual DCT setting

Participants sat at a table with 90 cm distance to a Dell P2213LED Display (22 inches display diagonal) that we used as an output device. The display ran on a resolution of 1680 × 1050 px (refresh rate 60 Hz). An ordinary QWERTZ keyboard was located in front of the participant as an input device. In each trial, a custom-made C++ program presented two dot patterns on the screen, one on the left side and one on the right side. The dots of the left pattern were placed randomly in a range of 1/8 to 3/8 in width and 1/4 to 3/4 in height of the screen size. The dots of the right pattern were placed randomly in a range of 5/8 to 7/8 in width of the screen size with the same range of height as the left pattern. Dots varied randomly in 40% of diameter around the base diameter size of 10 pixel per dot, which is between ~0.33° and 0.65° diameters in visual angle (participants visual angle: ~17°).

We presented dot patterns in two different ways that allowed us to control for different possible confounding variables (see Halberda et al., 2008). In the average dot size control condition (DSC, 50% of trials), the dots in both patterns had on average the same diameter. The disadvantage of this otherwise favorable feature is that the displayed dot patterns differ in their cumulative surface area on the screen (more pixels for the more numerous set). Therefore, the other 50% of all trials were surface area controlled (SAC), which ensures that the total pixel count of the two dot patterns on the screen were equal.

#### Visual DCT procedure

Each trial in the visual DCT started with a white fixation cross on black background. When the participant manually initiated the trial by a button press, the fixation cross vanished and two white dot patterns appeared upon black background. After 200 ms, the dot patterns disappeared and the whole screen got flushed with a white mask (300 cd/m^2^) to inhibit possible visual aftereffects. Participants responded with the correspondent arrow keys (left or right) which dot pattern contained a higher number of dots. Then the next trial started. Participants completed 40 practice trials in each of the sessions and 720 experimental trials subsequently (18 repetitions of blocks of 40 trials = 5 ratios × 2 versions × 2 visual control × 2 side of higher number). In each repetition, we shuffled the order of trials randomly.

#### Haptic DCT setting

**Fig. 1:**
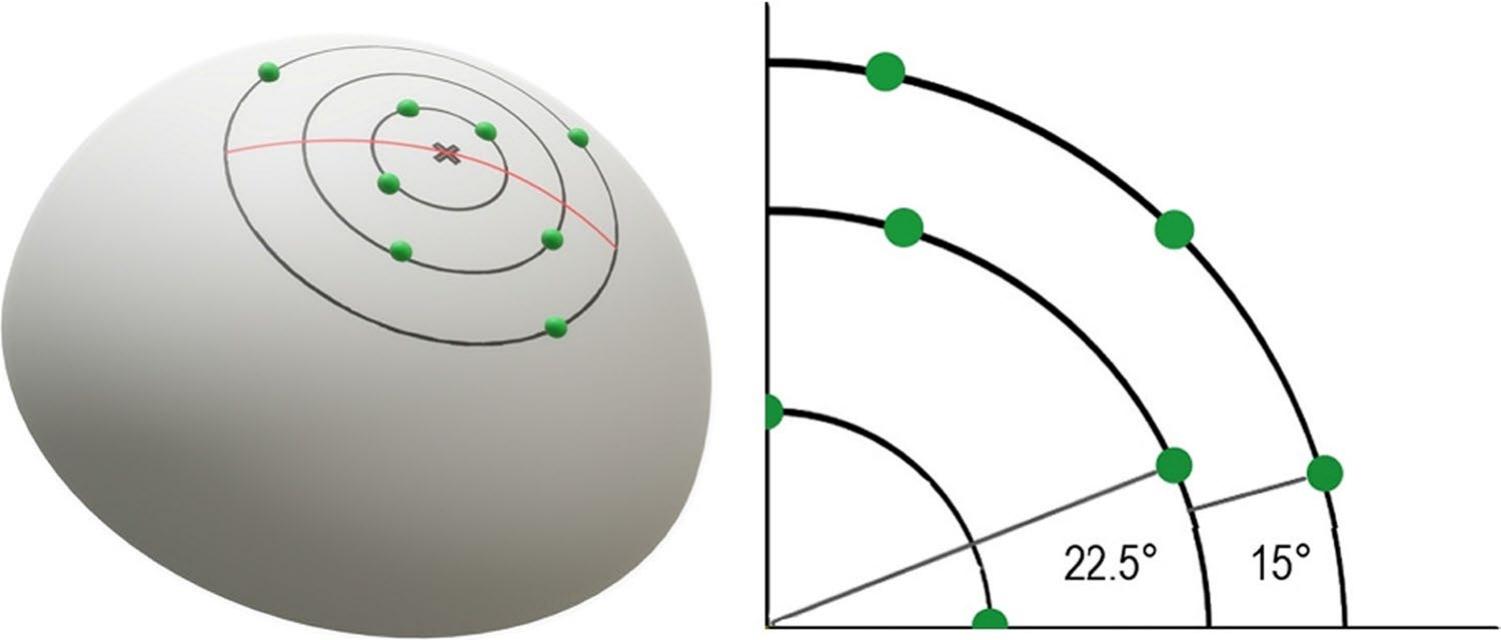
Left side: Styrofoam hemisphere with exemplary dots (pins) on three radial orbits. Right side: Quadrant with all possible slots for a pin. Position of the slots in degrees from the origin for one quadrant. Slots in the first orbit are perpendicular to the origin

Participants sat at a table behind an opaque curtain, which prevented the participant from seeing the stimuli during the whole experiment. The experimenter sat opposite to the participant on the other side of the curtain and exchanged the stimuli according to a predefined random trial list. We presented dot patterns in form of pins on Styrofoam hemispheres (industrial map pins of 5 mm in diameter each; see Fig. 1). The hemispheres were 150 mm in diameter and 75 mm in height. We coated them with jersey fabric to ensure a plain and pleasant surface. We marked three radial orbits (radii 15, 30, and 45 mm) onto the fabric in order to systematically organize the pins and capture their position. We centered the orbits on the “pinnacle” of the hemispheres. The inner orbit contained four slots, the intermediate eight slots, and the outer 12 slots for pins. The slot arrangement asserted that pins never formed a straight line, and that the distance between pins was always bigger than 11 mm. We conducted pilot tests (*N* = 5) to find the correct spacing, which allows to accurately individuate every single pin and minimizing the occupied area for convenient exploration. We found that a spacing of 11 mm between dots allowed a participant to reliably individuate a single pin in the palm of the hand (i.e., no errors without time limit). This result is consistent with that of Craig and Lyle (2001) who showed that different grating spaces are reliably discriminated when the spacing is set to 11 mm.

We preconfigured the stimuli for each participant and each session (i.e., we filled the slots on the hemispheres with pins according to a custom written C++ program that randomly chose positions on the hemisphere; two per 5 ratios × 2 versions = 20 hemispheres). In all single version hemispheres, we placed pins to slots of the inner and the intermediate orbit. In the double version hemispheres, we used all three orbits to enhance stimulus variety. We presented the hemispheres of each of the 10 pairs in two different orientations (0° and 120°) in order to increase the diversity of perceived pin configurations. In the haptic task, all pins were of the same size, so the haptic task can be considered an analogue to the visual dot-size control (DSC) condition.

#### Haptic DCT procedure

A haptic DCT trial started when the experimenter had placed the two Styrofoam hemispheres for exposure and the participant had stated to be ready. Five seconds of exploration time started when the palms of their hands touched the two stimuli. We chose the 5 seconds exploration time to ensure that participants can only approximate numerosity, as previously demonstrated in Ginsburg and Pringle (1988). We additionally validated that a participant’s counting performance for 10 pins in 5 seconds is at chance level in our own pilot data. The participants examined two hemispheres simultaneously with the palms of their hands. The experimenter rigorously observed the haptic exploration process, monitored the time limit with a stopwatch and announced when the time limit elapsed. After the exploration time expired, the experimenter asked the participant to raise hands and stop exploring the stimuli. The participant responded verbally with “left” or “right” which side contained more dots. If the experimenter had noticed that a participant did not touch all pins on the hemisphere or exceeded the time limit, the trial was repeated later in the experiment. In each session, participants initially examined a sample hemisphere with equipped pins, and then practiced the task in two trials. Afterwards, participants completed 80 experimental trials (5 ratios × 2 versions × 2 orientations × 2 side of higher number × 2 repetitions), which were ordered randomly.

#### Modality specific DCT adaptations

Our haptic and visual DCT share essential features of the general paired task paradigm, number ratios and the DSC. We, however, also made adaptations to account for particularities of modalities. First, the presentation time of stimuli differs between haptic and visual DCT (cf. Gimbert et al., 2016): Pilot investigations had shown that participants require a substantially longer time to extract the (“numerosity”) information with the palms of their hands as compared with the visual DCT, and we hence set haptic exploration time to 5 s. In contrast, extending the presentation time for the visual DCT toward the haptic time in the visual condition would result in participants being able to serially count dots. Thus, to tap similar processes in both tasks, we needed to use different presentation times.

Second, we used different visual dot sizes, but we did not use pins of different size in the haptic condition, nor did we implement a haptic analogue to the visual surface-area control condition. This is because the relation between pin size and haptic stimulation is different than that between dot size and visual stimulation: Haptic stimulation intensity relates to the extent of local skin deformation (Hayward, 2011), meaning that smaller pins will cause more intense stimulation than larger ones, and intensity further depends on pin shape and skin site. Thus, a straightforward transfer of the SAC was not possible, and the effects of different pin sizes would not have been well controlled. Still, the haptic task can be considered analogue to the visual DSC condition.

Third, due to different requirements of the DCTs (e.g., different interdot distances; visual discrimination of dots only requires an interdot distance as low as 0.28 mm), the overall necessary area, which a dot pattern occupies, as well as the overall possible density of the dot patterns, produces different variances in the convex hull ratio between the modalities. The relative convex hull of two dot patterns within a trial could vary from a minimum ratio of 1 to a maximum factor of .012 in the visual DCT and from a minimum ratio of 1 to a maximum factor of .183 in the haptic DCT. Our protocol generated “truly” random dot patterns for both modalities within their area boundaries and protocol restrictions (like interdot distance) resulting in different yet comparable and, due to a large number of trials, balanced range across the convex hull ratio scale (see Table 2).

**Table 2:** Descriptive statistics of the convex hull ratio for each DCT (visual condition 48,240 trials; haptic condition 10,720 trials)

Convex hull ratio	Min	1st Quantile	*Md*	*M*	3rd Quantile	Max	*SD*
Visual DCT	0.01	0.60	0.76	0.72	0.88	1.00	0.17
Haptic DCT	0.18	0.68	0.83	0.78	0.91	1.00	0.20

Last but not least, the number of trials was much higher in the visual as compared with the haptic task, because haptic trials take longer than visual trials. Reducing the number of visual trials to that of haptic trials might have increased parallelism between the tasks, but in our view, a better visual measurement was preferred.

Overall, despite and partly by means of these modality-specific adaptations, our design is well-suited to answer the question of whether the main source of a participant’s percept is indeed a number. In addition, our approach utilizes statistical methods proposed by DeWind et al. (2015), which allow us to determine a statistically controlled performance index that mainly reflects the ANS acuity (see Data Analysis section).

### Data analysis

For each participant, sensory modality, version, and session, we calculated the mean percentage correct per ratio. We used the mean percentage of correct answers to investigate the ratio dependency and to scrutinize the data for deviance in control conditions and then calculated Weber fractions. We excluded participants’ data if their overall mean percentage correct deviated more than three standard deviations from the sample mean in any of the sessions in any modality. Four of the 71 participants met this exclusion criterion. We then aggregated participants’ mean percentage correct over sessions, and submitted arcsine transformed (cf. Cohen et al., 2015, pp. 240–241) values into a repeated-measures analysis of variance (ANOVA), with the within-subjects variables ratio, version, and modality. We applied Greenhouse–Geisser correction (Geisser & Greenhouse, 1958) in case of sphericity violations.

To investigate the association between sensory modalities and task reliability, we used Weber fractions (*w*) as ANS acuity measure (see Equation 3). We calculated group *w*s for each sensory modality (visual/haptic) collapsing trials over all sessions, separate group *w*s for trials of the visual control condition DSC and SAC, and individual *w*s for each participant using trials of the two sessions (in the respective modality) separately to estimate task reliability, and for both sessions combined in order to study cross-modality correlations. Here, we additionally excluded individuals if their *w* deviated more than three standard deviations from the sample mean of the respective condition. This led to a partial exclusion of participants (up to three) in some visual conditions.

Further, we used the method proposed by DeWind et al. (2015) to quantify the contribution of different stimulus features in a DCT to the responses of participants. The method models the probability of choosing the stimulus that is placed on one particular side (here, the right side), as a function of influence factors that contribute to the percept of number. We pooled the response data over all participants for each modality (separate for the visual controls DSC and SAC). For the response model, influence factors on the number percept are estimated via a regression model in form of regression weights. We adapted Equation 7 in DeWind et al. (2015). The probability to choose the right side is then given by the equation:

1 $$\kern0.5em p\left( choose\ right\right)=\frac{1}{2}\ \left(1+\mathit{\operatorname{erf}}\left(\frac{\log_2\left({r}_{num}\right)-\left(\mu \right)}{\sqrt{2}{\beta_{num}}^{-1}}\right)\right),$$

where *μ* is given by:

2 $$\mu =\frac{-{\beta}_{side}-{\beta}_{spacing}\ {\log}_2\left({r}_{spacing}\right)}{\beta_{num}}.$$

In Equations 1 and 2, *erf* is the Gaussian error function, *β*_*num*_ and *β*_*spacing*_ are the regression weights for the number ratio and spacing ratio, respectively, and *β*_*side*_ reflects a bias towards a side. The number ratio *r*_*num*_ is calculated by dividing the number of dots on the right side by the number of dots on the left side (log-values then range from −1, which means that on the left side dots are twice as numerous, to 1, which means dots are twice as numerous on the right side). The spacing ratio predictor *r*_*spacing*_ is a ratio of sparsity (convex hull divided by number of dots on the respective side) times the convex hull area for the respective sides. Regression parameters *β*_*num*_, *β*_*spacing*_, *β*_*side*_ in Equations 1 and 2 are obtained by fitting a generalized linear model (iteratively reweighted least squares) via a probit link function of the response variable (0 = left side, 1 = right side) with log_2_ transformed predictors of number ratio and spacing ratio.

The regression weights themselves indicate a relative contribution of the factor to the number percept and therefore should be identical for each modality, if the exact same processes determined the number percept. To test relevant regression coefficients between the models for significant differences we utilized a *z*-test procedure proposed by Clogg et al. (1995).

We applied the methods used to estimate the overall model’s regression weights also to each individual data set in order to estimate the individual regression coefficient *β*_*num*, *i*_. From this coefficient, we determined each participant’s Weber fraction *w*_*i*_ as a measure of ANS acuity using the following equation:

3 $$w=\frac{1}{\sqrt{2\kern0.5em }{\beta}_{num}}.$$

For a detailed description of how to prepare the predictors and an in-depth explanation of the model, we refer to DeWind et al. (2015). As a brief summary, we use the regression model to quantify the influence of spacing and number to the numerosity percept in both modalities and use resulting individual *w*’s for a correlation of performance across modalities; for reliability estimates and group *w*s for an overall evaluation of performance between modalities.

We used R (Version 3.6.1; R Core Team, 2019) for all data analyses and utilized the ggplot2 library (Wickham, 2016) for data visualization.

## Results

### Ratio dependency

**Fig. 2:**
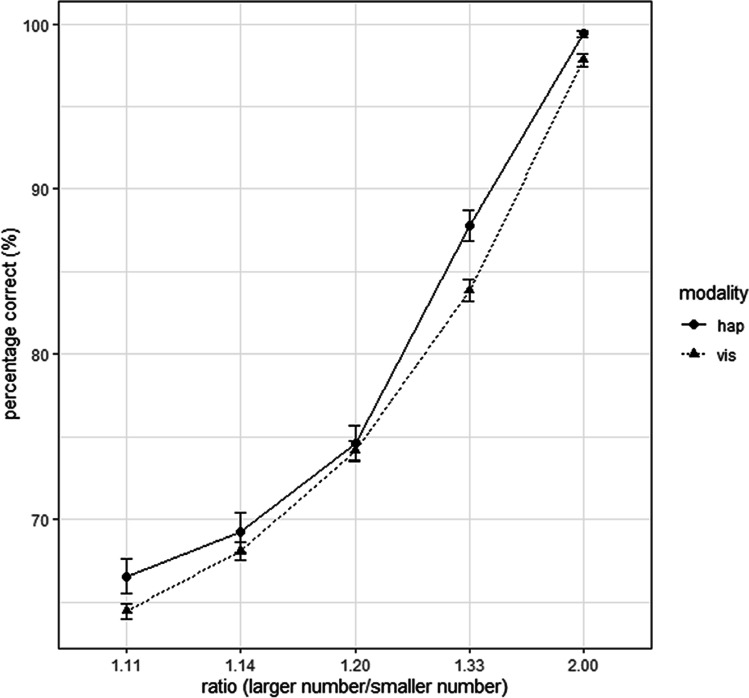
Ratio-to-correct responses plot for visual and haptic DCTs aggregated over sessions (semi log plot). Error bars represent the standard error of the mean

Table 3 lists the aggregated mean percent correct of all 67 participants sorted by number ratio and session. Figure 2 shows the average percentage correct responses for each modality and number ratio, aggregated over both sessions.

**Table 3:** Relative frequencies of correct trials (*M*) and standard deviations (*SD*) of 67 participants sorted by number ratio, session (t), and visual and haptic DCT

		Ratio									
		2.00		1.33		1.20		1.14		1.11	
		*M*	*SD*	*M*	*SD*	*M*	*SD*	*M*	*SD*	*M*	*SD*
t1											
	Visual	.977	.032	.831	.056	.741	.053	.678	.050	.642	.046
	Haptic	.991	.027	.876	.095	.756	.116	.680	.139	.665	.101
t2											
	Visual	.979	.042	.846	.067	.742	.056	.684	.058	.647	.050
	Haptic	.997	.013	.880	.096	.736	.129	.704	.134	.666	.129

The repeated-measures ANOVA (rmANOVA) with the variables number ratio (2.00, 1.30, 1.20, 1.14, and 1.11) and sensory modality (haptic, visual) revealed a significant main effect of ratio, *F*(4, 264) = 1367.56, *p* < .001, ${\upeta}_p^2$ = .954. All repeated contrasts of adjacent levels of the variable ratio were significant, all *p*s < .001 (2.00 vs. 1.33, 1.33 vs. 1.20, 1.20 vs. 1.14, 1.14 vs. 1.11) after Bonferroni adjustment. Participants showed in general more correct responses for higher ratios (see Fig. 2). Also, the main effect of modality was significant, *F*(1, 66) = 17.81, *p* < .001, ${\upeta}_p^2$ = .212. Participants performed overall better in the haptic DCT compared with the visual DCT, *M*_*hap*_ = 79.5% (*SD* = 4.10) to *M*_*vis*_ = 77.7% (*SD* = 3.71). The Ratio × Modality interaction was significant, *F*(4, 264) = 7.39, *p* < .001, ${\upeta}_p^2$ = .101. Interaction contrasts between pairs of successive levels of factor ratio show that the interaction is driven by comparison 1.33 versus 1.20, *F*(1, 66) = 9.32, *p* = .003, other interaction contrasts turned out not to be significant.

We also compared the “single” and the “double” version for each modality. The rmANOVAs with the variables number ratio (2.00, 1.33, 1.20, 1.14, and 1.11) and version (single, double) revealed a significant main effect of ratio, *F*(4, 264) = 404.77, *p* < .001, ${\upeta}_p^2$ = .860 (haptic) and *F*(4, 264) = 1751.29, *p* < .001, ${\upeta}_p^2$ = .964 (visual), but not of version neither in the visual, *F*(1, 66) = 0.24, *p* = .629, ${\upeta}_p^2$ = .004 (*M*_single_ = 77.6%, *M*_double_ = 77.8%), nor in the haptic modality, *F*(1, 66) = 1.79, *p* = .186, ${\upeta}_p^2$ = .026 (*M*_single_ = 80.1%, *M*_double_ = 78.9%). The Ratio × Version interaction was not significant neither in the visual, *F*(4, 264) = 0.96, *p* = .424, ${\upeta}_p^2$ = .014, nor in the haptic modality, *F*(4, 264) = 1.76, *p* = .153, ${\upeta}_p^2$ = .026. This indicates ratio dependency because the total amount of dots is not a significant factor.

Furthermore, we conducted three Bonferroni adjusted (corrected for three comparisons) rmANOVAs with one variable always being number ratio and the other variable being the control condition in the visual modality (DSC/SAC), session in the haptic modality or session in the visual modality. Any of these three analyses showed a significant factor number ratio exclusively, *p* < .001, but no significant differences in the respective control condition or session variable, nor interaction effects, except of a significant main effect in the visual control condition DSC versus SAC with *F*(1, 66) = 40.35, *p* < .001, ${\upeta}_p^2$ = .379 (*M*_*DSC*_ = 78.7%, *SD* = 3.57; *M*_*SAC*_ = 76.6%, *SD* = 4.29). Even though differences in the visual controls turned out to be significant, we aggregated the trials of the respective conditions for the correlational analysis because individual ANS acuity indicators (*w*) for SAC and DSC correlated strongly with each other, *r*(66) = .871, *p* < .001. For the response modeling however, we treated the visual conditions separately to contrast them against the haptic responses and investigate their differences.

### Correlational analysis and reliability

**Fig. 3:**
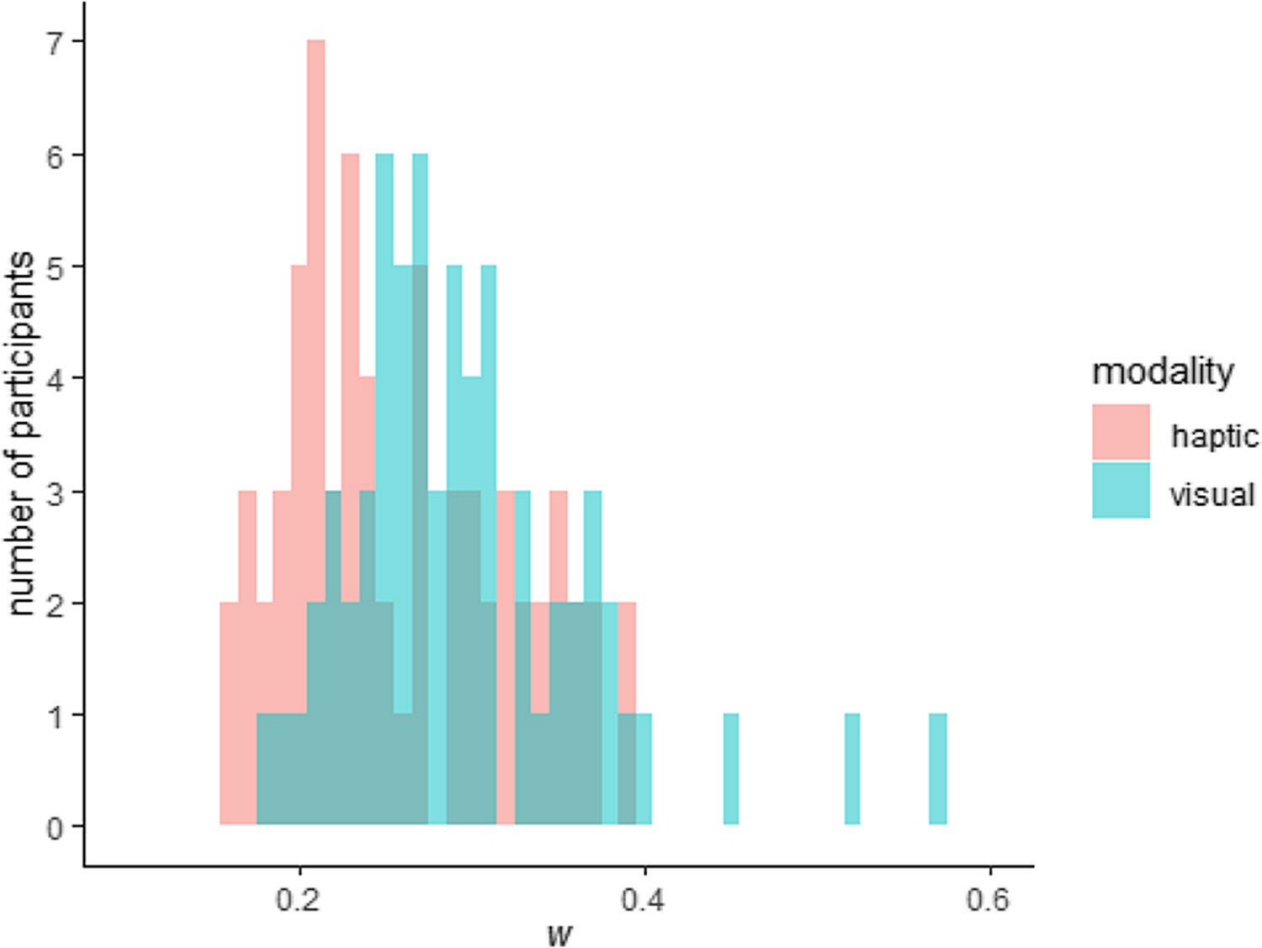
Distribution of individual *w*s (ANS acuity) in the visual and haptic DCTs. Outlier values are not depicted

The histogram in Fig. 3 shows the distribution of the individual Weber fractions (*w*) per sensory modality fitted over both sessions.

Table 4 shows the correlations for individual *w*’s between sessions per sensory modality as well as the correlations between the two modalities (trials aggregated over sessions). From intramodal correlations between sessions we calculate the Spearman–Brown adjusted reliability (Brown, 1910) coefficients, because these take into account that the complete haptic and visual tasks (from which we calculate cross-modal correlations) are based on trials from both sessions. Other statistics in Table 4 belong to the nonadjusted values.

**Table 4:** Correlations of ANS acuity (*w*) across individuals, test statistics, and adjusted task reliability (ATR)

Modality	*r*	*N*	*t*	*df*	CI 95% [lower]	CI 95% [upper]	*P*	*ATR*
haptic–haptic	.251	67	2.088	65	0.011	0.463	.041*	.401
visual–visual	.736	64	8.549	62	0.598	0.831	<.001**	.848
haptic–visual	−.133	65	−1.067	63	−0.365	0.114	.290	–

*Note*. Intramodal correlations are calculated between first and second session; cross-modal correlations are calculated between modalities (trials aggregated over both sessions, respectively). Spearman–Brown corrected reliability (for the number of trials in the cross-modal correlation) is reported. Statistics calculated for 80 trials.

**Fig. 4:**
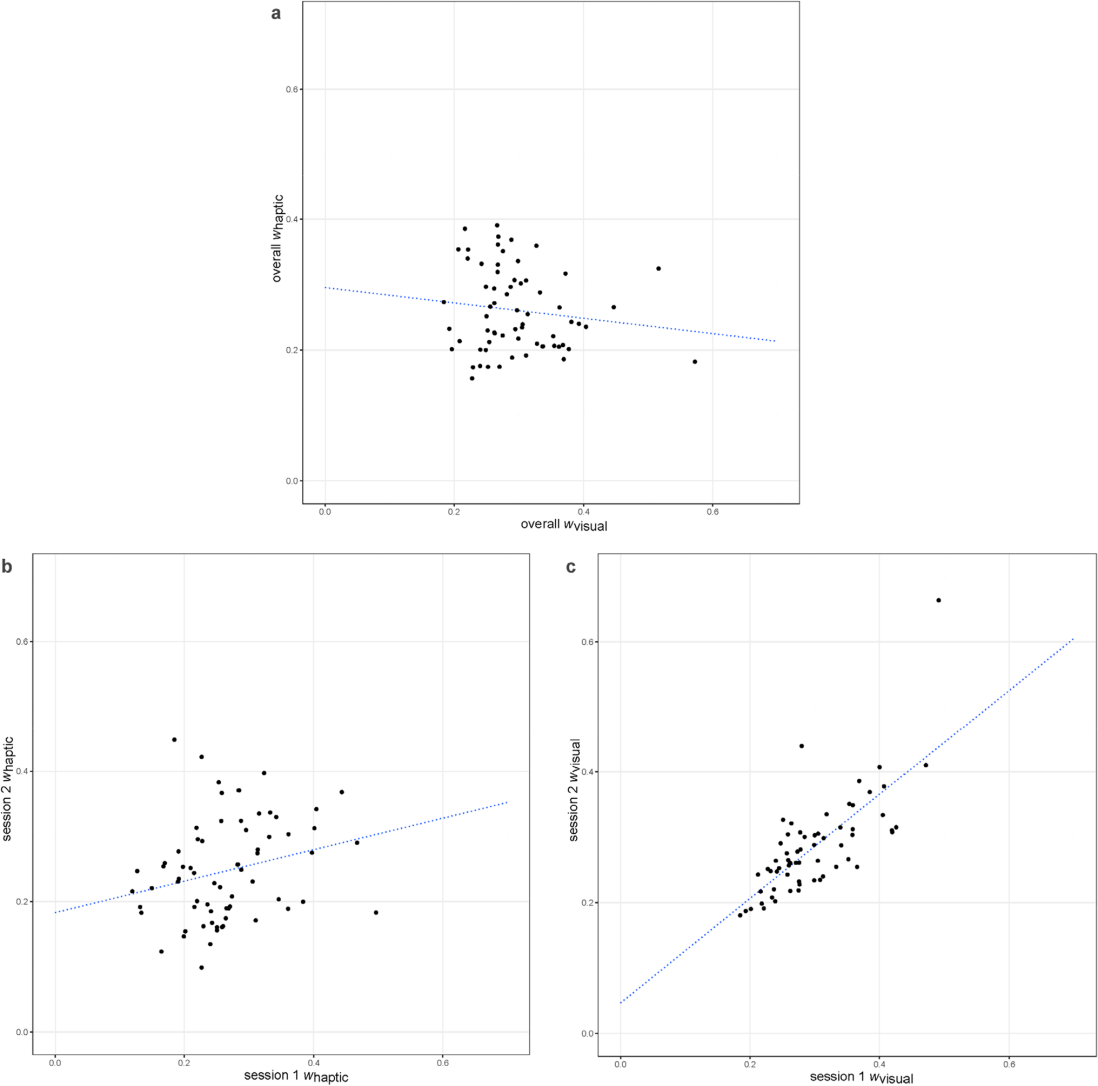
Scatterplots of individual *w*’s (ANS acuity) for the visual and the haptic DCTs. **a** visual–haptic, **b** haptic t1–t2, and **c** visual t1–t2. Intramodal correlations are calculated between first and second session; cross-modal correlations are calculated between modalities (aggregated over both sessions; t1 = Session 1, t2 = Session 2, respectively)

Figure 4 shows the scatterplots for each of the three correlations shown in Table 4.

The visual DCT shows with an intersession-correlation of *r*(62) = .736, *p* < .001 an acceptable adjusted task reliability of .848. The correlation coefficient within the haptic DCT shows a smaller but significant association, *r*(65) = .251, *p* = .041, implying a task reliability of .401. The cross-modal performance coefficient does not indicate statistically significant correspondence, *r*(63) = −.133, *p* = .290. It is noteworthy that the confidence interval of the cross-modal correlation does not cover the case of a medium-sized positive correlation effect of ρ = .3, rejecting the hypothesis that there is a medium-sized or larger correlation between haptic and visual individual performance. One may argue that the suboptimal haptic reliability may have obscured the true association between modalities. However, when applying a correction for attenuation (Spearman, 1904) in order to estimate correlations

4 $$\frac{r_{v,h}}{\sqrt{Rel_v}\ast \sqrt{Rel_h}}\kern0.5em$$

without measurement error, the values still reject the hypothesis of a medium-sized or larger true positive correlation (95% CI after applying attenuation correction to its upper and lower borders: −0.449 to 0.018).

### Modality specific models of numerosity comparison

We modeled the response behavior of participants (data collapsed over participants) for the visual and the haptic dataset according to Eqs. 1 and 2. This allowed us to calculate the probability to choose the right side for both modalities as a function of number, spacing-related features and side bias. We investigated DSC and SAC controlled trials from the visual DCT separately. Table 5 shows statistics of log_2_-transformed predictors.

**Table 5:** Descriptive statistics of the log_2_-transformed predictors we used for the logistic regression for each modality (visual condition 48,240 trials, haptic condition 10,720 trials)

Predictor (*log*_2_)		Min	1st Quantile	*Md*	*M*	3rd Quantile	Max
Haptic							
	*r*_*num*_	−1.00	−0.26	0.00	0.00	0.26	1.00
	*r*_*spacing*_	−3.90	−0.39	0.00	0.00	0.39	3.90
Visual_DSC_							
	*r*_*num*_	−1.00	−0.26	0.00	0.00	0.26	1.00
	*r*_*spacing*_	−11.74	−0.68	0.00	0.00	0.67	9.94
Visual_SAC_							
	*r*_*num*_	−1.00	−0.26	0.00	0.00	0.26	1.00
	*r*_*spacing*_	−9.67	−0.68	0.00	0.00	0.67	9.33

*Note. r*_*num*_ refers to number ratio, *r*_*spacing*_ to spacing ratio. DSC = average dot sized controlled visual condition; SAC = surface area controlled visual condition.

**Fig. 5:**
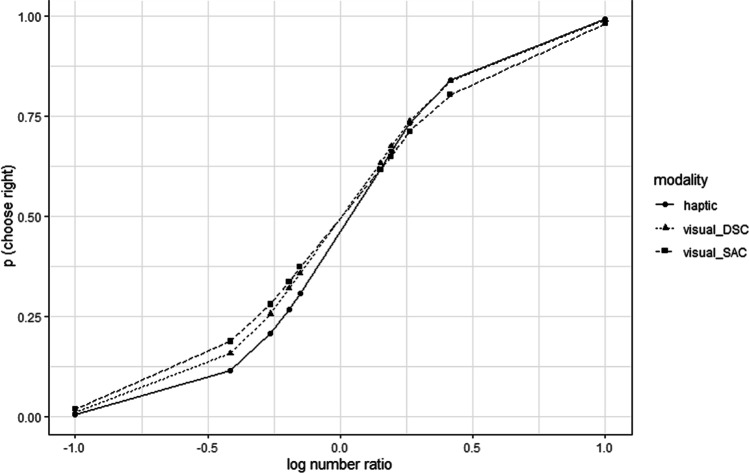
Probabilities of choosing the right side differentiated by the log_2_ ratio of the two compared numerosities (number ratio). Log number ratio of zero indicates that the two dot patterns are equal in number

The results of the visual models (each *n*_visual_ = 48,240 trials) and haptic model (*n*_haptic_ = 10,720 trials) to predict (on group level) the response probabilities to choose the right side dependent on numerosity are depicted in Fig. 5. The model statistics are given in Table 6.The corresponding estimated regression weights are given in Table 7. The statistics show similar and reasonably good pseudo *R*^2^ indices (cf. McFadden, 1977) across all three models. The AICs differ between models, which can be attributed to differences in the total amount of sample trials between modalities.

**Table 6:** Model statistics of the logistic regression for each modality (visual condition 48,240 trials, haptic condition 10,720 trials)

Model	AIC	*Null deviance*	pseudo *R*-squared
Haptic	9411.6	14832.8	0.365
Visual_DSC_	42914	66872	0.358
Visual_SAC_	45531	66869	0.319

*Note.* AIC = Akaike´s Information Criterion. McFadden Pseudo *R*-squared. DSC = average dot sized controlled visual condition; SAC = surface area controlled visual condition.

**Table 7:** Estimated model coefficients and statistics for coefficient tests against 0 for each modality (visual condition: 48,240 trials; haptic condition: 10,720 trials)

Coefficient		*β*	*Z*	*SE*	*p*
Haptic					
	*β*_*num*_	2.534	48.39	0.052	<.001***
	*β*_*spacing*_	0.264	10.38	0.025	<.001***
	*β*_*side*_	−0.101	−6.82	0.015	<.001***
Visual DSC					
	*β*_*num*_	2.349	103.462	0.023	<.001***
	*β*_*spacing*_	0.267	41.917	0.006	<.001***
	*β*_*side*_	−0.010	−1.457	0.007	.145
Visual SAC					
	*β*_*num*_	2.045	101.537	0.020	<.001***
	*β*_*spacing*_	0.270	43.620	0.006	<.001***
	*β*_*side*_	−0.017	−2.568	0.007	.010^a^

*Note. β*_*num*_ and *β*_*spacing*_ are regression weights for the *log*_2_ number and spacing ratios, respectively, and *β*_*side*_reflects a response bias towards one side; *z* is the test statistics of a significance test against zero, *SE* is the standard error of the regression weights.

^a^ not significant after Bonferroni adjustment.

We compared the regression weights *β*_*num*_ and *β*_*spacing*_ of number ratio (*r*_*num*_) and spacing ratio (*r*_*spacing*_), respectively, between the three models (DSC, SAC, haptic) in multiple Bonferroni-adjusted *z* tests (Clogg et al., 1995) There are no significant differences between the coefficients *β*_*spacing*_: *z*_*haptic/DSC*_ = −0.116, *p* = .452; *z*_*haptic/SAC*_ = −0.232, *p* = .492; and *z*_*DSC/SAC*_ = −0.341, *p* = .488. However, the coefficient *β*_*num*_ was significantly higher for the haptic as compared with the visual models, *z*_*haptic/DSC*_ = 3.244, *p* < .001, and *z*_*haptic/SAC*_ = 8.716, *p* < .001, and it was higher for visual DSC as compared with the visual SAC model, *z*_*DSC/SAC*_ = 10.011, *p* < .001. Correspondingly (cf. Equation 3), the group Weber fraction *w* (i.e., the estimate for the average ANS acuity) indicates the best ANS acuity for the haptic modality, *w*_*haptic*_ = .28, and the worst for the visual SAC condition, *w*_*DSC*_ = .30, *w*_*SAC*_ = .35.

## Discussion

This study investigated nonsymbolic number processing in the haptic and visual modality by means of a dot comparison task. We evaluated if there is evidence for a shared cognitive system (ANS), which processes number independent of the sensory modality. We modeled the performance in the DCT in both modalities on an individual and a group level and evaluated if the measurement is (a) reliable and (b) if performance in both modalities is associated with one another. Our results show that regardless of whether stimuli were examined by touch or by vision, the responses depended on the number ratio of dots between the presented dot patterns, but not on the absolute number of dots. This is a clear indicator of ratio dependency in both modalities. Our group regression model revealed that in the haptic as well as in the visual modality, the ratio of the compared numbers had the primary, major influence on the participants’ responses. However, the influence of this predictor slightly but significantly differed between the sensory modalities. Additionally, we found for both modalities a similarly sized smaller influence for less abstract, spacing-related factors (driven by convex hull), which is consistent with previous findings in the visual domain (Clayton et al., 2015). Only in the haptic modality, an additional tendency to choose a side (the left) regardless of the presented stimuli turned out to be a significant predictor but with a rather small effect. These results convincingly show that number ratio is the major influencing factor in both modalities when comparing numerosities of dot arrays in a DCT. However, they do not necessarily show that number processing is shared between the sensory modalities. The influence of number slightly differed between modalities, giving room for speculation that modality-specific mechanisms might better account for these results. We cannot give a definite answer as to what the exact mechanism is. Nonetheless, there definitely is additional significant influence on the “numerosity” percept, which consequently compromises the strong interpretation of the ANS theory. Even more importantly, we were not able to find a direct correlation between the individual Weber fractions of the haptic and the visual DCT, which would have indicated strong evidence for a modality-shared underlying system processing numerical information. We expected a medium-sized effect between the modalities, which, according to our analysis, is out of the confidence interval boundary. Given that number is the predominant feature involved in the processing of numerosity, the finding that we can reject a medium or stronger relation between haptic and visual DCT performance suggests that number is processed at least partly differently in the visual as compared with the haptic modality.

The ANS theory suggests that numerosity can be extracted from any given stimulus material and is not bound to a modality (Brannon & Merritt, 2011; Dehaene, 2011; Dehaene & Changeux, 1993; Izard et al., 2009; Tokita & Ishiguchi, 2016). In a strong interpretation of this theory, number is an independent feature, and the cognitive system (ANS) is able to filter out all confounding features accompanied with the stimulus material. In conclusion, ANS acuity of participants should be similar across modalities, at least to a high extent. Our results are certainly not consistent with a strong claim of ANS theory. Indeed, we found converging evidence for both the haptic and the visual modality that performance is ratio dependent and that number is the primary source when comparing dot arrays of DCTs. A closer look, however, shows that performance scores were slightly elevated in the haptic condition. This may reflect subtle differences in the DCT implementation of the visual and haptic tasks, or it may indicate factual differences in processing. In addition, the effect of number ratio differed between modalities and even within a modality (i.e., comparing regression coefficients of the visual controls DSC and SAC), and factors beyond number ratio contribute to the responses. Differences between modalities and between visual conditions became visible when we predict the probability of choosing a side (see Fig. 5). For a mutually shared system only extracting numerosity, predictions should have been highly similar (cf. Tokita et al., 2013). A broader interpretation of the ANS would allow for additional factors other than number ratio to play a role. Smaller modality differences in ratio-dependent performance are in principle in line with a broader interpretation of a shared ANS system. However, we can further reject a medium or stronger association between individual ANS acuity in the two modalities, which also contrasts a broader interpretation of the assumption of a single underlying system encoding numerosity in a modality-independent manner.

Our conclusions contradict the interpretations of the conceptual and methodological closest study by Gimbert et al. (2016). The major difference is that we did not accept ratio-dependency as the sole indicator for the involvement of a shared ANS when comparing two different modalities. We think that our enhancements in the haptic stimulus material, and particularly in data analysis, offer supporting evidence for the assumption of a common cognitive system for numerosity encoding between the visual and haptic modality were made precipitously. We think that before numerosity judgments in both modalities can be put into a common framework, substantial differences we found here (i.e., different numerosity weights accompanied by nearly identical spacing weights and lack of cross-sensory associations of individual performance) have to be integrated into a coherent or adapted theory first. In line with results of Clayton et al. (2015), DeWind et al. (2015), and DeWind and Brannon (2016), we suggest that spacing-related factors like convex hull, in addition to number, influence the percept as well. In addition to the already mentioned factors that contribute to the numerosity percept, general cognitive mechanisms, e.g., attention or inhibitory control, may also affect or even explain an association between performances of DCTs (cf. Anobile et al., 2020; Malone et al., 2019). This implies that if an association of performance indices between a visual and a haptic DCT can be found, it will be challenging and important to exactly identify what enabled the comparison between modalities and rule out potential broader mechanisms. Regarding this, we want to emphasize two more aspects, which we think are important to cover in future experiments similar to ours. The first is that participants might (unintentionally) use certain strategies during a task (e.g., use the shape of a pattern as proxy for the numerosity), which can be applied for both modalities and bypass the actual mechanism of interest (i.e., numerosity processing), and therefore compromising results. A way to address this would be to systematically assess participants strategies in the haptic DCT (cf. Dietrich et al., 2019) and generate balanced stimulus patterns (pin patterns), which are less prone to the (effective) use of the most frequent strategies. Another aspect is that participants might respond to a nonnumerical, quantity related dimension of the pins (e.g., surface area). In this case, associations across modalities can be even reliable but reflect quantity comparison rather than numerosity comparison. To examine this further, it could help to clarify the specificity of numerosity estimation in the haptic modality in contrast to a quantity estimation task. This could be achieved with a haptic control task, conceptually similar to a quantity estimation task from Leibovich and Henik (2014), in which participants compare shapes of varying area in a two alternative forced choice task. A resulting performance index in such a task could be used to clarify whether a shared variance to the performance of the numerosity comparison task exists. These, amongst other possible aspects, have to be systematically explored further to improve experiments using visual and especially haptic DCTs.

Overall, we think there is much evidence to shift the perspective from a concept of a shared numerical system that is solely accountable for number estimation to a more integrative system that acknowledges recent findings (Barth et al., 2003; DeWind et al., 2015; DeWind & Brannon, 2016; Gebuis et al., 2016; Leibovich et al., 2017; Smets et al., 2014), rather than to dwell on not having measured the “pure ANS” construct by focusing on methodological details, which occurred in the past. We think that if the ANS is not robust enough to encode numerosities across reasonably similar settings, as, is the case in our study, it is feasible to change the theoretical perspective accordingly.

Different authors were arguing that the ANS theory in its core is not sufficient anymore and needs either revision, for instance a more differentiated approach of quantity processing (Leibovich et al., 2017), or a complete shift in perspective on quantity estimation (Gebuis et al., 2016). For example, Gebuis et al. (2016) argue that ANS theory struggles to explain behavioral as well as neurophysiological data in several instances and a more general system they call “sensory integration system” would better account for reoccurring phenomena regarding confounds. The sensory integration theory differs in two essential points from the ANS theory: (a) sensory cues are integrated into the percept rather than filtered out and (b) the resulting estimation is not an abstract (i.e., directly comparable) number (Gebuis et al., 2016). Following that idea, any “confounding” variable would have weight in the percept and number is estimated by an overall integration of the stimulus features. Taking this theoretical perspective, it would be almost implausible that a “number comparison task” like our DCTs in different modalities and with stimulus features, where only number is kept constant and other potentially equally important features determine the estimate, would result in converging percepts. Taking this into consideration and the fact that number itself seems to be perceived differently between modalities, the lack of correspondence between DCTs in our experiment is comprehensible.

The field of ANS-research generally focuses on group-level comparisons (Halberda et al., 2008; Park & Brannon, 2013; Price et al., 2012), just as we did here. However, this may neglect that individuals differ in their perceptual processes (e.g., perceptual speed), which may be orthogonal to the numerosity comparison acuity. An adjustment for individual processing differences will allow for a better decision whether or not a “truly amodal” ANS exists. In this work, we focused mainly on matching the DCTs between modalities. Although, a part of matching the affordances of the DCTs in both modalities required some specific adjustments which led to task differences (e.g., presentation times, spacing ranges), one may wonder whether this itself can provide an alternative explanation for the absence of performance associations between modalities. Even slight task-variations within a modality can result in significant performance differences as exposed by our visual_SAC_ and visual_DSC_ conditions. Our approach of calculating Weber fractions with the DeWind formula seems favorable to us, as it accounts statistically for implementation differences. Nevertheless, it is important to notice that Weber fractions as performance indices themselves are not a guarantee to have an abstract comparable index for numerosity comparison acuity devoid of context (Guillaume & van Rinsveld, 2018). However, it allowed us to estimate influence of different features to the percept and deviations within a paradigm can at least partly be compensated if sufficient performance indices are used (DeWind & Brannon, 2016), like we did.

Another point is that even though we increased the number of trials in our study to a reasonable degree, it has not been enough to get a good reliability for the individual performance in the haptic DCT. DeWind and Brannon (2016) pointed out that poor correlations are possible because of a lack of reliability in only one dot comparison task (DeWind & Brannon, 2016). This gives room to speculate whether a correlation between modalities might have become visible if the haptic DCT had been more reliable. We acknowledge this possibility to a certain degree. However, the haptic DCT showed some consistency between sessions and an attenuation projection to account for minor reliability in the cross-modal correlation reveals that a medium sized positive correlation effect can be statistically rejected. We take the liberty of noting that our study did check reliability issues and only hence allows taking lower reliability into consideration, which elsewhere has been ignored at all. Nevertheless, we acknowledge the rather low reliability of the haptic DCT as a shortcoming, which impedes a fully drawn conclusion that a shared numerosity system between vision and haptics is unlikely. A higher reliability of the haptic DCT would have been desirable to dispel doubts from this side. In future work, to overcome shortcomings with reliability, we would recommend an increase in trials. Reliability generally increases with an increase of experimental trials and high trial numbers in DCTs are highly recommended (Dietrich et al., 2015; Lindskog et al., 2013). Further studies should investigate if it is possible to update a haptic DCT to achieve similar reliability as visual DCTs. Another recommendation is to further converge and consolidate task specifications between the DCTs of the visual and the haptic modality. This concerns, for example, overall variety of dot patterns in the haptic modality, which a participant examines within an experiment. Fully randomized dot patterns in every trial (a quite laborious approach) would allow confirmation of performance differences we found between modalities. Another important refinement would be to include and systematically vary the variable area in the haptic DCT to which we were agnostic here. We think that a clean implementation of such a control condition requires further investigation and is even topic for several separate studies because it is possible that a direct transfer from the visual DCT to the haptic DCT setting is not consequently appropriate or applicable in active touch. An additional implementation of these factors in a model like the one in the current study could give deeper insights into the mechanisms that contribute to the numerosity percept in the haptic modality.

## Conclusion and outlook

The current study contributes two major findings to the field: Firstly, strong claims of the ANS theory (i.e., number is strictly independent from other features) are not supported by our data. We doubt that neither with preceding reported haptic DCTs nor with our haptic or visual DCT, participants would respond only to number during the task. Secondly, the claim of an amodal shared ANS is also not supported by our data, given that visual and haptic number estimation performances were not related, at least not moderately or more. Following the idea of a sensory integration system, it will be a challenging but important task to disentangle the relevant factors that are integrated into the numerosity percept in the haptic modality. Further research is needed to clarify what the actual similarities are in the visual, haptic, or the auditory modality integration. For now, we think it is feasible to assume that humans can encode numerosity from haptic stimulus material with additional influence of number-related features, which seem to be similar but not equivalent in both the haptic and the visual modality.

## References

[CR1] Anobile G, Arrighi R, Castaldi E, Grassi E, Pedonese L, Moscoso PAM, Burr DC (2018). Spatial but not temporal numerosity thresholds correlate with formal math skills in children. Developmental Psychology.

[CR2] Anobile G, Castaldi E, Moscoso PAM, Burr DC, Arrighi R (2020). "Groupitizing": A strategy for numerosity estimation. Scientific Reports.

[CR3] Anobile G, Cicchini GM, Burr DC (2016). Number As a Primary Perceptual Attribute: A Review. Perception.

[CR4] Barth H, Kanwisher N, Spelke E (2003). The construction of large number representations in adults. Cognition.

[CR5] Barth H, La Mont K, Lipton J, Spelke ES (2005). Abstract number and arithmetic in preschool children. Proceedings of the National Academy of Sciences of the United States of America.

[CR6] Bertamini M, Zito M, Scott-Samuel NE, Hulleman J (2016). Spatial clustering and its effect on perceived clustering, numerosity, and dispersion. Attention, Perception & Psychophysics.

[CR7] Brannon, E. M., & Merritt, D. J. (2011). Evolutionary foundations of the Approximate Number System. In *Space, time and number in the brain* (pp. 207–224). Elsevier. 10.1016/B978-0-12-385948-8.00014-1

[CR8] Brown W (1910). Some experimental results in the correlation of mental abilities. British Journal of Psychology, 1904–1920.

[CR9] Butterworth B (2010). Foundational numerical capacities and the origins of dyscalculia. Trends in Cognitive Sciences.

[CR10] Clayton S, Gilmore C, Inglis M (2015). Dot comparison stimuli are not all alike: The effect of different visual controls on ANS measurement. Acta Psychologica.

[CR11] Clogg, C. C., Petkova, E., & Haritou, A. (1995). Statistical methods for comparing regression coefficients between models. Advance online publication. 10.1086/230638

[CR12] Cohen J, Cohen P, West SG, Aiken LS (2015). *Applied multiple regression/correlation analysis for the behavioral sciences*.

[CR13] Craig JC, Lyle KB (2001). A comparison of tactile spatial sensitivity on the palm and fingerpad. Perception & Psychophysics.

[CR14] Dehaene, S. (2011). *The number sense: How the mind creates mathematics* (Rev. and updated ed.). Oxford University Press. http://gbv.eblib.com/patron/FullRecord.aspx?p=716741

[CR15] Dehaene S, Changeux JP (1993). Development of elementary numerical abilities: A neuronal model. Journal of Cognitive Neuroscience.

[CR16] DeWind NK, Adams GK, Platt ML, Brannon EM (2015). Modeling the approximate number system to quantify the contribution of visual stimulus features. Cognition.

[CR17] DeWind NK, Brannon EM (2016). Significant Inter-Test Reliability across Approximate Number System Assessments. Frontiers in Psychology.

[CR18] Dietrich, J. F., Huber, S., & Nuerk, H.-C. (2015). Methodological aspects to be considered when measuring the approximate number system (ANS)—A research review. *Frontiers in Psychology*, *6*, 295. 10.3389/fpsyg.2015.00295 10.3389/fpsyg.2015.00295 PMC4362052 25852612

[CR19] Dietrich JF, Nuerk H-C, Klein E, Moeller K, Huber S (2019). Set size influences the relationship between ANS acuity and math performance: A result of different strategies?. Psychological Research.

[CR20] De Smedt Bert, Noël Marie-Pascale, Gilmore Camilla, Ansari Daniel (2013). How do symbolic and non-symbolic numerical magnitude processing skills relate to individual differences in children's mathematical skills? A review of evidence from brain and behavior. Trends in Neuroscience and Education.

[CR21] Faul F, Erdfelder E, Buchner A, Lang A-G (2009). Statistical power analyses using G*Power 3.1: Tests for correlation and regression analyses. Behavior Research Methods.

[CR22] Feigenson L, Dehaene S, Spelke E (2004). Core systems of number. Trends in Cognitive Sciences.

[CR23] Gebuis T, Cohen Kadosh R, Gevers W (2016). Sensory-integration system rather than approximate number system underlies numerosity processing: A critical review. Acta Psychologica.

[CR24] Geisser S, Greenhouse SW (1958). An extension of box's results on the use of the *F* distribution in multivariate analysis. The Annals of Mathematical Statistics.

[CR25] Gimbert F, Gentaz E, Camos V, Mazens K (2016). Children's Approximate Number System in haptic modality. Perception.

[CR26] Ginsburg N, Pringle L (1988). Haptic numerosity perception: Effect of item arrangement. The American Journal of Psychology.

[CR27] Guillaume M, van Rinsveld A (2018). Comparing numerical comparison tasks: A meta-analysis of the variability of the Weber fraction relative to the generation algorithm. Frontiers in Psychology.

[CR28] Halberda J, Mazzocco MMM, Feigenson L (2008). Individual differences in non-verbal number acuity correlate with maths achievement. Nature.

[CR29] Hayward V (2011). Is there a 'plenhaptic' function? *Philosophical Transactions of the Royal Society of London*. Series B, Biological Sciences.

[CR30] Hyde DC (2011). Two systems of nonsymbolic numerical cognition. Frontiers in Human Neuroscience.

[CR31] Inglis M, Gilmore C (2014). Indexing the approximate number system. Acta Psychologica.

[CR32] Izard V, Sann C, Spelke ES, Streri A (2009). Newborn infants perceive abstract numbers. Proceedings of the National Academy of Sciences of the United States of America.

[CR33] Jordan NC, Kaplan D, Nabors Oláh L, Locuniak MN (2006). Number sense growth in kindergarten: A longitudinal investigation of children at risk for mathematics difficulties. Child Development.

[CR34] Leibovich T, Henik A (2014). Comparing performance in discrete and continuous comparison tasks. Quarterly Journal of Experimental Psychology (2006).

[CR35] Leibovich T, Katzin N, Harel M, Henik A (2017). From "sense of number" to "sense of magnitude": The role of continuous magnitudes in numerical cognition. The Behavioral and Brain Sciences.

[CR36] Lindskog M, Winman A, Juslin P, Poom L (2013). Measuring acuity of the approximate number system reliably and validly: The evaluation of an adaptive test procedure. Frontiers in Psychology.

[CR37] Malone SA, Pritchard VE, Heron-Delaney M, Burgoyne K, Lervåg A, Hulme C (2019). The relationship between numerosity discrimination and arithmetic skill reflects the approximate number system and cannot be explained by inhibitory control. Journal of Experimental Child Psychology.

[CR38] McFadden, D. (1977). *Quantitative methods for analyzing travel behaviour of individuals: Some recent developments*. Cowles Foundation Discussion Papers (No. 474). Retrieved from Cowles Foundation for Research in Economics, Yale University website: https://EconPapers.repec.org/RePEc:cwl:cwldpp:474

[CR39] Mou Y, vanMarle K (2014). Two core systems of numerical representation in infants. Developmental Review.

[CR40] Nieder A (2016). The neuronal code for number. Nature Reviews. Neuroscience.

[CR41] Olsson L, Östergren R, Träff U (2016). Developmental dyscalculia: A deficit in the approximate number system or an access deficit?. Cognitive Development.

[CR42] Park J, Brannon EM (2013). Training the approximate number system improves math proficiency. Psychological Science.

[CR43] Piazza M (2010). Neurocognitive start-up tools for symbolic number representations. Trends in Cognitive Sciences.

[CR44] Price GR, Palmer D, Battista C, Ansari D (2012). Nonsymbolic numerical magnitude comparison: Reliability and validity of different task variants and outcome measures, and their relationship to arithmetic achievement in adults. Acta Psychologica.

[CR45] R Core Team (2019). R: A language and environment for statistical computing [Computer software].

[CR46] Smets K, Gebuis T, Defever E, Reynvoet B (2014). Concurrent validity of approximate number sense tasks in adults and children. Acta Psychologica.

[CR47] Spearman C (1904). The proof and measurement of association between two things. The American Journal of Psychology.

[CR48] Szkudlarek, E., & Brannon, E. M. (2017). Does the approximate number system serve as a foundation for symbolic mathematics? *Language Learning and Development: The Official Journal of the Society for Language Development*, *13*(2), 171–190. 10.1080/15475441.2016.1263573 10.1080/15475441.2016.1263573 PMC5362122 28344520

[CR49] Szűcs D, Myers T (2017). A critical analysis of design, facts, bias and inference in the approximate number system training literature: A systematic review. Trends in Neuroscience and Education.

[CR50] Tokita M, Ashitani Y, Ishiguchi A (2013). Is approximate numerical judgment truly modality-independent? Visual, auditory, and cross-modal comparisons. Attention, Perception, & Psychophysics.

[CR51] Tokita M, Ishiguchi A (2016). Precision and Bias in Approximate Numerical Judgment in Auditory, Tactile, and Cross-modal Presentation. Perception.

[CR52] Tomlinson RC, DeWind NK, Brannon EM (2020). Number sense biases children's area judgments. Cognition.

[CR53] Wickham, H. (2016). ggplot2: Elegant graphics for data analysis [Computer software]. https://ggplot2.tidyverse.org

[CR54] World Medical Association Declaration of Helsinki: Ethical principles for medical research involving human subjects (2013). *JAMA*, *310*(20), 2191–2194. 10.1001/jama.2013.281053 10.1001/jama.2013.281053 24141714

